# Making Career in Dentistry. A City Practice

**Published:** 1904-03

**Authors:** Frank W. Sage

**Affiliations:** Cincinnati, O.


THE AMERICAN

### Journal of Dental Science.

### MAKING CAREER IN DENTISTRY. A CITY PRACTICE.

By FRANK W. SAGE, D.D.S., Cincinnati, O.

Every student matriculating at a dental college is fairly certain, sooner or later, to feel a longing for a city practice. A young man may have felt contented with the prospect of going back to the small town, village or hamlet whence he came, to practice, until he came to the dental college, saw and appreciated the advantages of a city practice, and so felt a stirring ambition to enroll his name among those of the leaders in the profession there established.

The dental college clinic likely enough, first suggests to the student the possibility of realizing this dream. In the promiscuous assembly of patients attending the clinics, the student sees not a few who appear the equals socially of the average patients thronging the office of his preceptor at home. Judging them by externals, he is apt to assume ability on their part to pay fairly good fees. Indeed, he may find by pushing a little a circle just outside of the clinic, friends of these patients who have no prejudices against a young dentist who does good work and who will patronize him and pay at least an average fee. Thus it comes about that before long he has picked up quite a little practice, more or less remunerative, out of college hours. From this small beginning he comes in time to ask himself, "Why shouldn't I get a paying practice right here in this city?"

Back of these reflections are considerations familiar to every student. The young man has heard the admonition, "If you want to make money you must go where money abounds." Doubtless he has visited the offices of half a dozen city dentists of various degrees, taking notes as he went. He has noted various obvious advantages of a city clientele; he assumes that city patients are more appreciative of fine dental services than their country cousins; pay more ungrudging and more promptly; are better trained to business obligations; keep their appointments better; notify the dentist if unable to keep them; and recognize more expressly the dentist's claim to a professional standing and professional consideration.

Other considerations as to the possibilities of superior office equipments come up: the availability of water, of electricity; the proximity of dental supply houses; the privilege of restricted hours, and of entertainment out of office hours. Add to these the elbow to elbow contact with leading representative men of the profession, the dignity of class assumed to attach to a city dentist simply by virtue of his being a city dentist (!) and the wonder is that the fever in the blood of every student in the dental college does not consume him—the fever of desire for a city practice.

The picture certainly is alluring, and if the truth were known, has tempted many an older head to leave a good country practice in order to benefit through the real or assumed advantages of a city practice.

"There are so many repelling features about a country practice," they will say. "Patients come with foul mouths to us, regarding us as no better than scavengers; they track mud into our reception rooms, perhaps bring a dog or two with them to help ruin our carpets. They have no modesty about questioning your prices, they assail your integrity by charging failure when anything goes wrong; they condescend to you, as if you of course prized their patronage above measure. At the best there is so much tinkering and fussing over little things that take no end of time, and yet that you are unable to charge for at all adequately, that one might as well be a cobbler and done with it."

It is no secret that dentists have been driven into other occupations by discouragements of this nature.

"There is no money in dentistry. I intend to give up practice, if I can find anything better," many have said, and are today saying. Numbers make a change, with varying experiences of success or failure, as we all know.

It may be premised in passing that the dentist who is not enough of a manager of his affairs to be able to devise ways for overcoming these peculiar difficulties encountered in practice, will, beyond doubt, be equally harrassed and annoyed by trifling obstacles in any calling he may choose to adopt.

Of course the first consideration presenting to any dentist, young or old, whose eyes may be turned cityward, concerns the matter of money. Everything is secondary and subordinate to that. A dentist will put up with serious annoyances, if paid enough. The mistake outsiders make is in assuming that city dentists escape the peculiar class of evils above described. Human nature is the same the world over, and the country dentist who is annoyed because a patient announced in the presence of a roomful of waiting patients that a filling has come out (while eating soup), will quickly learn that the most exalted of the city dentist's patients do the same thing.

Now beyond question it is true that many city dentists have their practice so organized and regulated that they escape or evade many trying experiences which daunt the country or town dentist. It is all in the man, however; let this statement be appreciated in the outset. Some dentists —some *men,* for it is all a question of personality—so impress themselves on their patrons that they always seem to hold the "upper hand," as the saying is. I am addressing the young men of the profession now, and cannot too much emphasize the truth that personal dignity, the exhibition of force of character, of honest, earnest purpose do more to repel little liberties of insolence and of impertinence which patients often practice on a dentist, than any special scheme, however ingenious, for impressing them with one's importance. Yet it is for the reason that certain classes of people require an outward show of importance and consequence before they will be impressed with respect for the dentist's personality that such artificial accessories as I shall now name are brought into requisition by certain dentists in cities of the first class particularly.

Paradoxical as it may seem, one of the first things a "swell" city dentist is apt to do, is to make himself somewhat difficult of access to his patients. He is willing to serve you—in your turn, and in case your demand on him is deemed of sufficient importance to claim a hearing. But you cannot rush up to him as you would to a window at the postoffice, throw down your money and back away with your stamps and change all within a few seconds. Oh, no. You call at his office, ring and are ushered in by a charming young lady. She is dignified, suave, attentive, yet you feel that you cannot rush past her and confront the doctor unannounced. Perhaps she refers you to another charmer, further back, provided she has not discovered that you want to sell the doctor a book. This guardian of the inner portal offers you a dainty card and pencil, inviting your superscription. The card then passes into the *sanctum sanctorum*, while you wait in the reception room. There you discover several earlier arrivals, waiting in silent patience. A solemn hush broods over the apartments; you perhaps wonder if you ought not to have tendered a fee in advance.

Red tape you call this. Of course it is. Everybody knows it is red tape, and many doubtless go away and poke fun at the doctor's spurious ceremony. But when they receive a bill seemingly out of proportion for the service rendered, they think again about the red tape and pay without protest. (There seems to be a tacit understanding that red tape must be paid for.) Those who cannot pay discover the fact in advance usually. A five hundred dollar "vawse" standing on a pedestal in the hall, and half a dozen fur rugs skating around on the hard-wood floor, make wholly unnecessary any inquiry as to fees.

A city dentist of this class is certain to gain a large following from the wealthy, provided he is master of his profession. He gets well paid for every service.

It would seem that any dentist with money to furnish all the accessories named, provided he be a gentleman in appearance at least, ought to be able to carry his part in such a scheme as this. Nothing is further from the truth. A man is born to this sort of thing. If not, he may dress his office and himself to the last degree, and yet fail. There is really a good deal of humbug about this external show, as the dentist himself doubtless knows. You say there's no particular knack in practicing humbuggery. Oh, isn't there! Do not be deceived; in nothing is more perfect consistency required. A single slip may undo you. Go to the wrong church a few times; carry a bundle along a fashionable thoroughfare; join the wrong golf club and see what happens!

Fashion in dentistry as in other matters is a jealous mistress, and must be served just so.

Next below these men, in point of grandeur, pomp and all that, comes the numerous class of dentists who put on more or less style, some more, others less. Numbers of these dentists imitate the methods of the very stylish men already described, yet fail to attain the high degree of success supposed to be theirs. Others, conscious of not being equal to such things, make no attempt in that direction. Would not if they could cultivate the petty arts of these men. It is after all largely a matter of taste. Some even declare they do not want the patronage of the very wealthy. They esteem the second grade of wealthy people, or even the fairly well-to-do, as better patients in all respects. It is an open question, of course.

You may enter one of these offices with less ceremony than is required of one visiting the very swellest offices. More or less red tape is discernible, of course. The man who uses none at all is rather apt to find himself at the mercy of numbers of well-meaning but pestiferous people who are heedlessly annoying in all the ways country patients are, and superadd a choice selection of peculiarly exasperating ways calculated to drive a dentist mad. For instance there is the woman who telephones to change her appointment, telephones later that she has reconsidered and will come, and winds up by coming three hours late, making you miss a train. Many good church members, Sunday school teachers, who ought to have learned to do to others as they would be done by, seem to forget that dentists deserve a share of such consideration. They would be fined ten dollars for each offense of the sort by some high-class dentists. But the poor dentist who has no system, who keeps on day after day hoping vainly that people will in time learn to be as considerate of his interests as he claims to be of theirs, will sink from one depth of humiliation to another, and it will never occur to him that he is wholly to blame, because he has never given his patients to understand that he has certain rules mutually of advantage to himself and his patients.

It is all in a lifetime, and some dentists seem just as happy and contented without any rules or system in their offices, and without any money, for that matter. Which is certainly very fortunate.

It is not necessary to subdivide these classes, as might be done. For all practical purposes as regards the question what may the young dentist expect who turns his face cityward, the enumeration of classes of professional men in dental practice there, with whom he must associate and probably compete, is complete enough. There are the advertisers with whom all other classes must compete. Rather, it may be said, against whom all other classes are arrayed. However sturdily the established "regulars" may disclaim competition with them, the newcomer can hardly disregard them in estimating his chances of gaining a practice. Many intelligent, self-respecting people do patronize the advertisers. It seems that an outsider may have no regard whatever for dentistry as a profession, and yet hold a fairly elevated position in society. Let us not be deceived. Professional men have come more and more, of late years, to depend for the respect and confidence of those out of the professions upon their individual qualities, not on the accident of association with others calling themselves as a body a profession. Which is as it should be.

Now the suggestion is, finally, to every student, to every established dentist elsewhere, pondering the matter of coming to a large city to practice, to place himself squarely before a mirror, look himself carefully over, and ask himself impartially, am I adapted to the requirements of a city practice? It seems there are the extremes of patronage for which a dentist who achieves much of a money success usually aims. The one is, the very wealthy, or, at least, the exclusives, refined, well-to-do people, who care something for style, and who go to a certain dentist because he is in a way exclusive, restricting his practice to their class. The other extreme is represented by the thousands of any class who may be drawn in by specious advertising and whose needs must be met by hiring a corps of assistants. The idea is, get the work done with dispatch, get the money, start in with another patient without loss of time. This practice is usually called a business. And a business it is.

To state the sum of the matter, a dentist coming to the city must be either a "business" dentist, or he must possess the somewhat artificial ways and devices of the ultra-fashionable, in order to make any considerable impression. Otherwise he might as well stay in the country. For the middle and lower grades of people who patronize a dentist at all, in the cities, are quite as likely to prove unprofitable to the dentist as are the average classes in smaller places, or in the country. It is safe to say there is a much larger class of people living from hand to mouth in cities, who at the same time seem to be fairly well-to-do, than the country affords.

The dental colleges are themselves a serious menace to any but the higher classes of dentists in any city. It cannot be longer denied. Many who once patronized the middle class man now go to the dental college clinic. It is the fashion to decry the quality of work done in the colleges, yet the fact remains that much of it is excellent.

So then this is to be considered by the student thinking of locating in the city. Let him not be too well assured that he will find it all smooth sailing. He might ofttimes do quite as well in the country, if he would try the experiment of putting on a little style and perhaps some restrictions. Human nature is the same the world over, or for all practical purposes we will say, "the United States over," and it seems necessary to guard against invasions of one's rights in every business and profession. You yourself, no doubt, often invade innocently even other peoples' rights. You perhaps enter a store, asking permission to leave a package awhile, and it never occurs to you that fifty others do the same thing at that same store every day. Fifty responsibilities thrust upon some clerk, each trifling, yet all taken together no doubt burdensome. And no suggestion of compensation.

The dentist in country or city needs to protect himself against people who think altogether of their own convenience, and really ignore his in an innocent way. They tell you to make the appointment for a certain hour, then casually suggest, as they go out, that if they fail to come then, expect them at the same hour the following day. Ignorance, thoughtlessness, nothing more. They want to see you only a moment and keep you half an hour; send word that the work required will take not over an hour, and when you see them you find it cannot be done at all. And so on. We all know.

The dentist must cultivate system, in city or country. Must fend people off, as it were. Not to their serious inconvenience, of course; anything that serves as a hint is sufficient. A fair play suggestion, it may be.

An attendant to stand between the dentist and callers pays for herself by the impression she makes. Appointment cards should be used by every dentist. Let your patients understand that you prefer not to make an appointment until you have seen what is required. Many absolutely decline to do it or exact a fee for the hour reserved, whether the full time is required or not. A professional friend said recently, "A dentist is either in the profession or out of it." (Meaning that there is no half way course.) If the dentist is part dentist, part politician, part anything else but dentist during office hours, he will be rather sure to neglect these little items pertaining to system and will come more and more to feel the annoyance of his patrons imposing on him.—*Items of Interest.*

